# Effect of Home Enteral Nutritional Support Compared With Normal Oral Diet in Postoperative Subjects With Upper Gastrointestinal Cancer Resection: A Meta-Analysis

**DOI:** 10.3389/fsurg.2022.844475

**Published:** 2022-02-18

**Authors:** Fang Liu, Xuling Pan, SuQing Zhao, RuiJun Ren, GuiXia Chang, Yu Mao

**Affiliations:** ^1^Department of Nursing, Eastern Hepatobiliary Surgery Hospital, Naval Medical University, Shanghai, China; ^2^General Surgery, The Second Affiliated Hospital of Soochow University, Suzhou, China; ^3^Health Management Center, The First Hospital of Hohhot, Hohhot, China; ^4^Interventional Department, The First Hospital of Hohhot, Hohhot, China; ^5^Thoracic Surgery, The First Hospital of Hohhot, Hohhot, China

**Keywords:** esophageal cancer, surgical removal, home enteral nutrition, oral diet, feeding related complications, hematological parameters, anthropometric measurements

## Abstract

**Introduction:**

We performed a meta-analysis to evaluate the influence of a home enteral nutritional support compared with a normal oral diet in postoperative subjects with upper gastrointestinal cancer resection.

**Methods:**

A systematic literature search up to December 2021 was done and 23 studies included 3,010 subjects with upper gastrointestinal cancer resection at the start of the study; 1,556 of them were given home enteral nutritional support and 1,454 were normal oral diet. We calculated the odds ratio (OR) and mean difference (MD) with 95% CIs to evaluate the influence of home enteral nutritional support compared with a normal oral diet in postoperative subjects with upper gastrointestinal cancer resection by the dichotomous or continuous methods with a random or fixed-influence model.

**Results:**

Home enteral nutritional support had significantly higher quality of life (MD, 2.08; 95% CI, 1.50–2.67, *p* < 0.001), better body weight change (MD, 1.87; 95% CI, 1.31–2.43, *p* < 0.001), higher albumin (MD, 1.27; 95% CI, 0.72–1.82, *p* < 0.001), and higher pre-albumin (MD, 30.79; 95% CI, 7.29–54.29, *p* = 0.01) compared to the normal oral diet in subjects with upper gastrointestinal cancer resection. However, home enteral nutritional support had no significant impact on the hemoglobin (MD, 4.64; 95% CI, −4.17 to 13.46, *p* = 0.30), and complications (OR, 1.03; 95% CI, 0.76–1.40, *p* = 0.83) compared to the normal oral diet in subjects with upper gastrointestinal cancer resection.

**Conclusions:**

Home enteral nutritional support had a significantly higher quality of life, better body weight change, higher albumin, and higher pre-albumin, and had no significant impact on the hemoglobin and complications compared to the normal oral diet in subjects with upper gastrointestinal cancer resection. Further studies are required.

## Background

Upper gastrointestinal cancer, mostly esophageal and gastric cancer, is the third most frequent cancer in the world, causing the second-highest cancer-associated mortality (1). Poor nutritional status is one of the chief reasons for high death in upper gastrointestinal cancer (2). Upper gastrointestinal cancer can decrease oral consumption, with up to 70% of subjects suffering clinically substantial weight loss at diagnosis (3). Standard management for upper gastrointestinal cancer, e.g., chemotherapy and surgery, worsen nutritional status (4). It is assessed that oral consumption is inadequate and could only satisfy up to 70% of the energy needed at discharge after upper gastrointestinal resection (5), joined with the alteration in usual diet patterns because of the gastrointestinal tract reconstruction, there is a high incidence of gastrointestinal problems in the first year after surgery (5). The latest meta-analysis showed a weight loss of up to 12% at 6 months postoperation, with over 50% of all subjects losing more than 10% body weight at 12 months after upper gastrointestinal resection (6). Concerning long-term results, up to 95% of subjects fail to recover the lost weight at 5 or more years after surgery (7), recommending that the initial weight loss after surgery has a persistent influence and that nutritional status might affect more adjuvant management. In addition, malnutrition is frequently followed by physical, psychological, and emotional symptoms, causing a decrease in quality of life (8). Guaranteeing nutritional support after hospital discharge is vital, but the best method of management remains indistinguishable. Guidelines for enhanced recovery after esophagectomy and gastrectomy suggest routine postoperative nutrition management, comprising enteral tube feeding or oral nutritional supplements (9, 10). Many studies lately have studied home enteral nutrition [home enteral tube feeding (11)] and oral nutritional supplements after hospital discharge for recovering the nutritional status of subjects with upper gastrointestinal cancer after hospital discharge (12–15). The European Society for Clinical Nutrition and Metabolism surgery working group considered both enteral tube feeding and oral nutritional supplements as enteral nutrition after gastrointestinal surgery and called them as nutritional management by the enteral route in the guideline (16). The influences of home enteral nutritional support are conflicting. Some of these studies revealed that home enteral nutritional support significantly improved the nutritional status of subjects compared with a normal oral diet (12–15). but other studies failed to show such improvement (17, 18). Though nutritional status improvement and living in a familiar environment with family members might improve quality of life, several studies have recommended that home enteral nutritional support might inflict subjects and their caregivers (19). Therefore, results involving quality of life varied. Moreover, outcomes regarding the safety of home enteral nutritional support were varying. Previously, there has been a lack of consensus about the best nutritional support program after hospital discharge after upper gastrointestinal resection. In 2019, the European Society for Clinical Nutrition and Metabolism established the first guideline on home enteral nutrition (20), concentrating on its methodology and clinical practice. Though the guidelines defined indications for home enteral nutrition, comprising gastrointestinal cancer subjects at risk of malnutrition, the exact influence of home enteral nutrition in upper gastrointestinal cancers has not been explained until now. Moreover, influence of the oral nutritional supplements after hospital discharge of subjects with upper gastrointestinal cancers is conflicting. Therefore, we performed the present meta-analysis to evaluate the effect of home enteral nutritional support compared with a normal oral diet in postoperative subjects with upper gastrointestinal cancer resection.

## Methods

This meta-analysis is organized according to the epidemiology statement (21), after the established methodology.

### Study Selection

The main objective of this study was to compare the influence of home enteral nutritional support compared with a normal oral diet in postoperative subjects with upper gastrointestinal cancer resection using the following tools, such as odds ratio (OR), frequency rate or relative risk, and CI of 95%.

**Figure 1:**
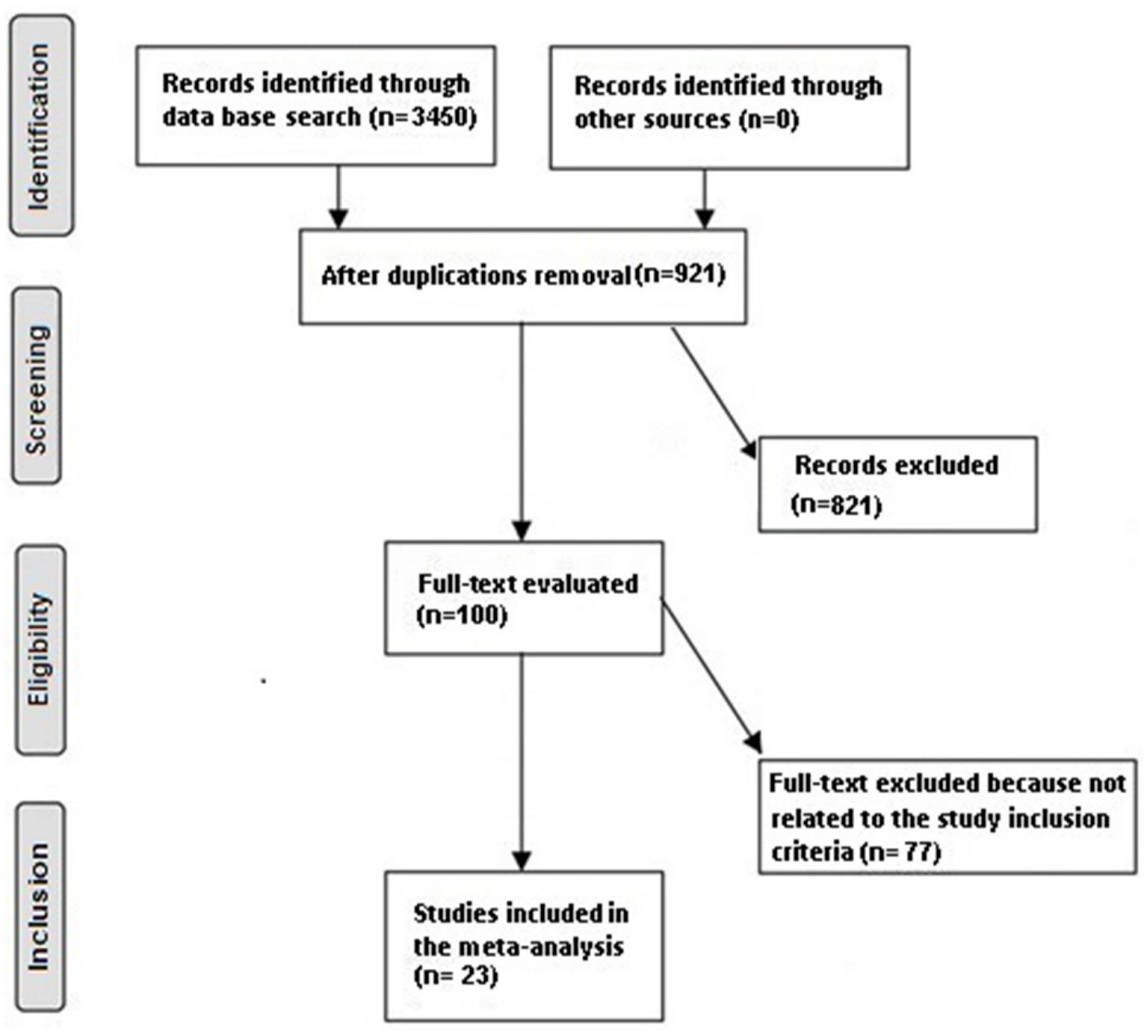
Schematic illustration of the study method.

The search was not narrowed to English, and inclusion criteria were not restricted by study type or size. Studies with no correlation were exempted from the study, e.g., editorials, review articles, letters, and commentary. Figure 1 exhibits the mode of analysis.

The article inclusion criteria were classified and integrated into the meta-analysis when:

1. The study was a randomized control trial, prospective study, or retrospective study.
2. The target population was subjected with upper gastrointestinal cancer resection.
3. The intervention program was home enteral nutritional support.
4. The study comprised comparisons between home enteral nutritional support and normal oral diet.

The next exclusion criteria were adopted among the intervention groups.

1. Studies that did not determine the influence of home enteral nutritional support compared with a normal oral diet in postoperative subjects with upper gastrointestinal cancer resection.
2. Studies with management other than home enteral nutritional support.
3. Studies that did not concentrate on the influence of comparative outcomes.

### Identification

PICOS principle was the protocol for the search strategy (22) and asserted the critical elements of PICOS as P (population): subjects with upper gastrointestinal cancer resection; I (intervention/exposure): home enteral nutritional support; C (comparison): home enteral nutritional support and normal oral diet; O (outcome): quality of life, bodyweight change, albumin, pre-albumin, hemoglobin, and complications; and S (study design), had no limitation (23). We conducted a systematic and brief search on MEDLINE/PubMed, Google Scholar, Embase, OVID, and Cochrane Library until December 2021, by a combination of keywords and correlated words for home enteral nutritional support, upper gastrointestinal cancer resection, normal oral diet, quality of life, bodyweight change, albumin, pre-albumin, hemoglobin, and complications as shown in Table 1. The selected studies were pooled in EndNote software to exclude the duplicates. Additionally, a thorough screening on the title and abstracts was done to erase any data that did not show any influence of home enteral nutritional support and normal oral diet on the outcomes studied for subjects with upper gastrointestinal cancer resection. Related pieces of information were collected from the remaining studies.

**Table 1:** Search strategy for each database.

**Database**	**Search strategy**
Pubmed	#1 “home enteral nutritional support” [MeSH Terms] OR “upper gastrointestinal cancer resection” [All Fields] OR “normal oral diet” [All Fields] #2 “quality of life” [MeSH Terms] OR “body weight change” [All Fields] OR “albumin” [All Fields] OR “pre-albumin” [All Fields] OR “hemoglobin” [All Fields] OR “complications” [All Fields] #3 #1 AND #2
Embase	‘home enteral nutritional support'/exp OR ‘upper gastrointestinal cancer resection'/exp OR ‘normal oral diet'/exp #2 ‘quality of life'/exp OR ‘body weight change'/exp OR ‘albumin'/exp OR ‘pre-albumin'/exp OR ‘hemoglobin'/exp OR ‘complications'/exp #3 #1 AND #2
Cochrane library	#1 (home enteral nutritional support):ti, ab, kw OR (upper gastrointestinal cancer resection):ti,ab,kw OR (normal oral diet):ti, ab, kw (Word variations have been searched) #2 (quality of life):ti, ab, kw OR (body weight change):ti, ab, kw OR (albumin):ti, ab, kw OR (pre-albumin):ti, ab, kw OR (hemoglobin):ti, ab, kw OR (complications):ti, ab, kw (Word variations have been searched) #3 #1 AND #2

### Screening

Subject-related and study-related data characteristics were considered for the collection and classification of data, and it was pooled into a standardized form. The categorization was made into the standard form, such as the surname of the first author, duration of the trial, place of practice, design of the study, subject type, sample size, categories, demography, treatment methodology, information source, method of evaluation (both qualitative and quantitative), statistical analysis, and primary outcome evaluation (22).

Methodological quality was assessed by the “risk of bias tool” adopted from Cochrane Handbook for Systematic Reviews of Interventions Version 5.1.0. This meta-analysis recommended that if a trial with inclusion criteria was based on the standards mentioned earlier, any conflicts that arose during the data collection by two reviewers were resolved through discussion and when necessary by the “corresponding author” to ensure the quality of the methodology (24).

### The Level of Risk of Bias Is Counted in the Assessment Criteria

The level of risk was considered low if all quality parameters were met. It was considered moderate if one of the quality parameters was not met/or partially met and was considered high if one of the quality parameters was not met/or not included. A re-examination of the original article was addressed for any inconsistencies.

### Eligibility Criteria

The main eligibility criteria concentrated on the influence of a home enteral nutritional support compared with a normal oral diet in postoperative subjects with upper gastrointestinal cancer resection. An evaluation of the influence of home enteral nutritional support and normal oral diet on the quality of life, body weight change, albumin, pre-albumin, hemoglobin, and complications in upper gastrointestinal cancer resection was conducted, and the data were extracted forming a summary.

### Inclusion

Studies reporting the influence of home enteral nutritional support compared with a normal oral diet in postoperative subjects with upper gastrointestinal cancer resection were only included in the sensitivity analysis. In comparison, the impact of home enteral nutritional support and normal oral diet cooperated were considered as a subcategory of sensitivity analysis.

### Statistical Analysis

The dichotomous or continuous methods were used to compute the OR and mean difference (MD) at a 95% CI on a fixed-influence or random-influence model. First, the I^2^ index range was established between 0 and 100%, when the I^2^ index scale for heterogeneity was indicated as no, low, moderate, and high as 0, 25, 50, and 75%, respectively (25). Random-influence was considered if I^2^ was >50%, and if <50%, as fixed-influence. The initial evaluation of the result was stratified, and in sub-group analysis, a *p*-value <0.05 was reported statistically significant. Egger regression test was used quantitatively and qualitatively to assess the publication bias (if *p* ≥ 0.05) by inspecting funnel plots of the logarithm of ORs compared with their SEs (22). The entire values of *p* were appeared two-tailed. The statistical analysis and graphs were done by “Reviewer Manager” version 5.3 (The Nordic Cochrane Center, The Cochrane Collaboration, Copenhagen, Denmark).

## Results

A total of 3,450 distinctive studies were found, of which 23 studies (between 2015 and 2021) satisfied the inclusion criteria and were comprised in the study (12–15, 17, 18, 26–41). This meta-analysis study based on 23 studies included 3,010 subjects with upper gastrointestinal cancer resection at the start of the study; 1,556 of them were given home enteral nutritional support and 1,454 were normal oral diet. All studies evaluated the influence of a home enteral nutritional support compared with a normal oral diet in postoperative subjects with upper gastrointestinal cancer resection. Ten studies reported data stratified to the quality of life; they all collected data using the same cancer-specific core questionnaire from the European Organization for Research and Treatment of Cancer, 18 studies reported data stratified to the bodyweight change, 9 studies reported data stratified to the albumin, 5 studies reported data stratified to the pre-albumin, 5 studies reported data stratified to the hemoglobin, and 9 studies reported data stratified to the complications. The study size ranged from 23 to 880 subjects with upper gastrointestinal cancer resection at the beginning of the study. The information of the 23 studies is shown in Table 2.

**Table 2:** Characteristics of the selected studies for the meta-analysis.

**Study**	**Country**	**Total**	**Home enteral nutritional support**	**Normal oral diet**
Bowrey et al. (12)	UK	41	20	21
Zhou et al. (13)	China	40	20	20
Xu et al. (14)	China	84	42	42
Gavazzi et al. (15)	Italy	69	34	35
Imamura et al. (26)	Japan	123	60	63
Hatao et al. (27)	Japan	113	64	49
Zeng et al. (28)	China	40	20	20
Ida et al. (17)	Japan	123	60	63
Froghi et al. (29)	UK	44	23	21
Ren et al. (30)	China	72	38	34
Cui et al. (31)	China	23	13	10
Hongyuan et al. (32)	China	50	25	25
Zhang et al. (33)	China	60	30	30
Kong et al. (34)	Korea	127	65	62
Liu et al. (18)	China	60	30	30
Liu et al. (35)	China	50	26	24
Li et al. (36)	China	62	30	32
Yang et al. (37)	China	315	200	115
Meng et al. (38)	China	337	171	166
Tan et al. (39)	China	212	105	107
Miyazaki et al. (40)	Japan	880	437	443
Yang et al. (41)	China	85	43	42
	**Total**	**3010**	**1556**	**1454**

**Figure 2:**
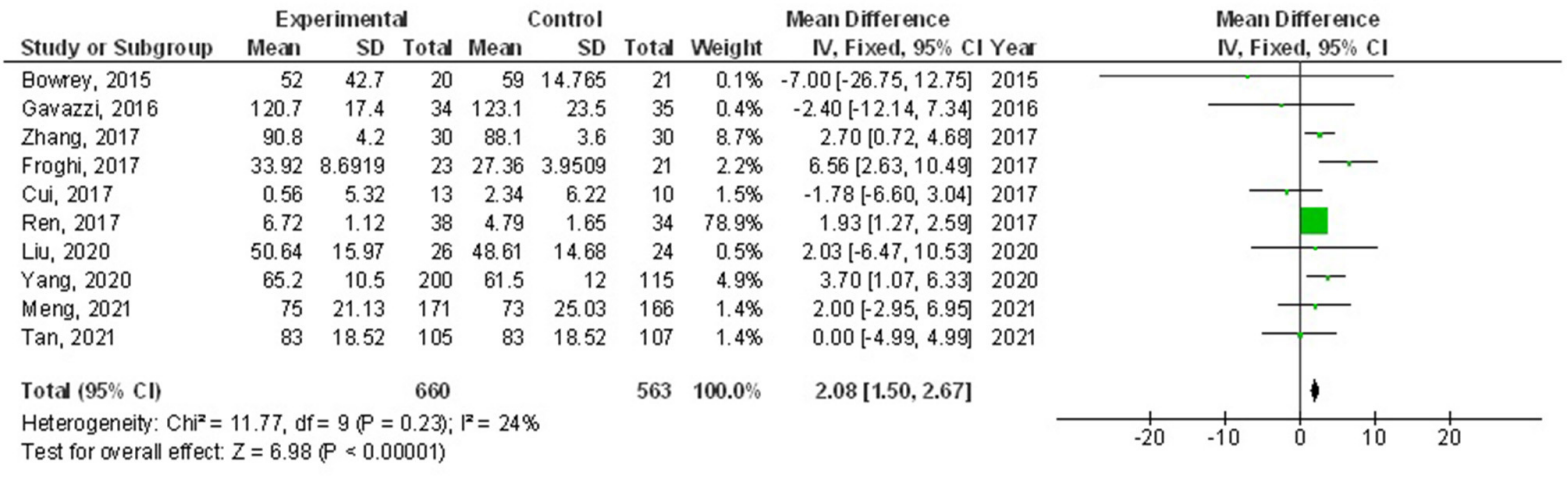
A forest plot of the quality of life in subjects with upper gastrointestinal cancer resection with the home enteral nutritional support compared to the normal oral diet.

**Figure 3:**
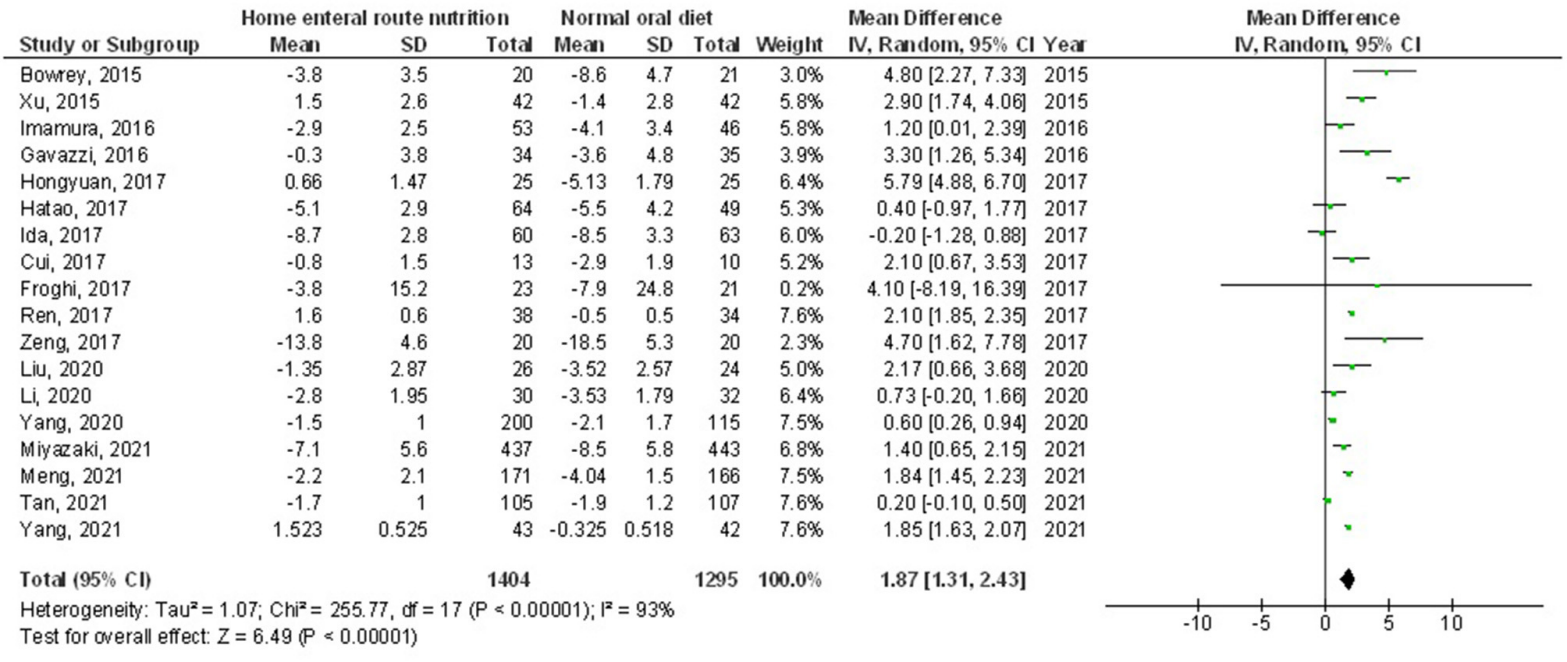
A forest plot of the bodyweight change in subjects with upper gastrointestinal cancer resection with the home enteral nutritional support compared to the normal oral diet.

**Figure 4:**
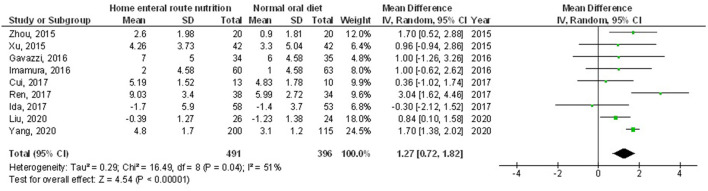
A forest plot of the albumin in subjects with upper gastrointestinal cancer resection with the home enteral nutritional support compared to the normal oral diet.

**Figure 5:**
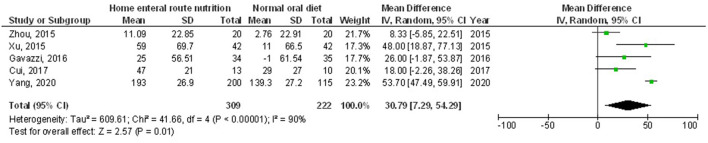
A forest plot of the pre-albumin in subjects with upper gastrointestinal cancer resection with the home enteral nutritional support compared to the normal oral diet.

**Figure 6:**
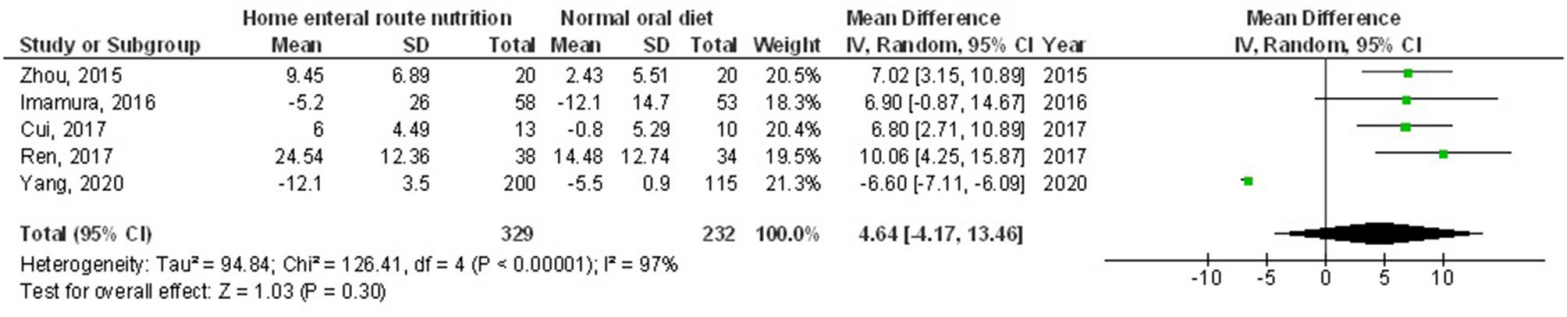
A forest plot of the hemoglobin in subjects with upper gastrointestinal cancer resection with the home enteral nutritional support compared to the normal oral diet.

**Figure 7:**
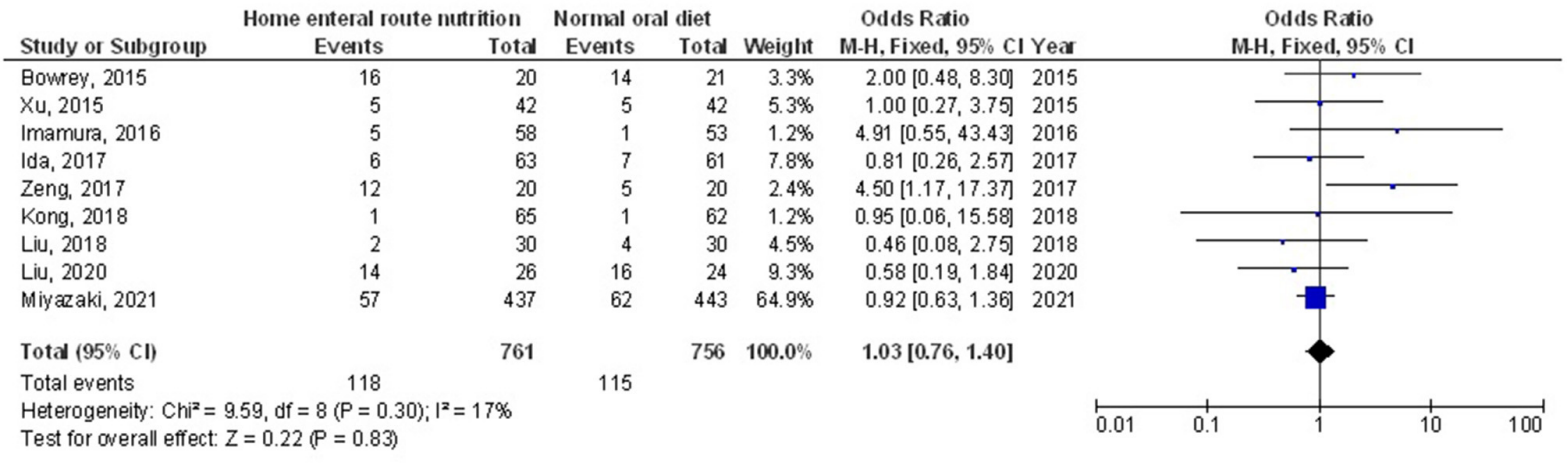
A forest plot of the complications in subjects with upper gastrointestinal cancer resection with the home enteral nutritional support compared to the normal oral diet.

Home enteral nutritional support had significantly higher quality of life (MD, 2.08; 95% CI, 1.50–2.67, *p* < 0.001) with low heterogeneity (I^2^ = 24%), better body weight change (MD, 1.87; 95% CI, 1.31–2.43, *p* < 0.001) with high heterogeneity (I^2^ = 93%), higher albumin (MD, 1.27; 95% CI, 0.72–1.82, *p* < 0.001) with moderate heterogeneity (I^2^ = 51%), and higher pre-albumin (MD, 30.79; 95% CI, 7.29–54.29, *p* = 0.01) with high heterogeneity (I^2^ = 90%) compared to a normal oral diet in subjects with upper gastrointestinal cancer resection as shown in Figures 2–5. However, home enteral nutritional support had no significant impact on the hemoglobin (MD, 4.64; 95% CI, −4.17 to 13.46, *p* = 0.30) with high heterogeneity (I^2^ = 97%) and complications (OR, 1.03; 95% CI, 0.76–1.40, *p* = 0.83) with no heterogeneity (I^2^ = 17%) compared to a normal oral diet in subjects with upper gastrointestinal cancer resection as shown in Figures 6, 7.

**Figure 8:**
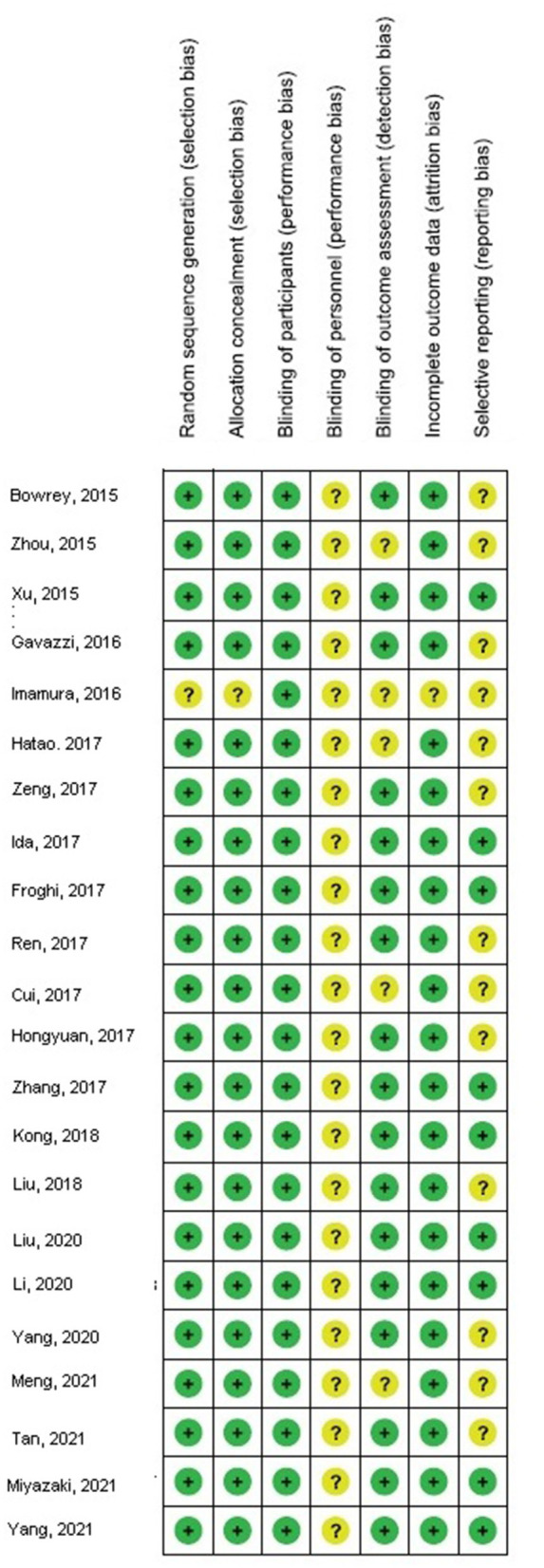
Risk of bias summary.

The stratified data did not examine factors, such as cost, age, gender, and ethnicity, between the two groups because no studies adjusted or outlined these factors. No publication bias (*p* = 0.89) was detected when the quantitative measurement was conducted using the Egger regression test and examination of the funnel plot. However, low methodological quality was observed in selected randomized control trials. No articles had selective reporting or incomplete data, which proved that selected articles were devoid of selective reporting bias as shown in Figure 8.

## Discussion

This meta-analysis study constructed on 23 studies included 3,010 subjects with upper gastrointestinal cancer resection at the start of the study; 1,556 of them were given home enteral nutritional support and 1,454 were normal oral diet (12–15, 17, 18, 26–41). Home enteral nutritional support had significantly higher quality of life, better body weight change, higher albumin, and higher pre-albumin compared to a normal oral diet in subjects with upper gastrointestinal cancer resection. However, home enteral nutritional support had no significant impact on the hemoglobin and complications compared to the normal oral diet in subjects with upper gastrointestinal cancer resection. However, the analysis of outcomes should be performed with consideration because of the low sample size of some of the selected studies found for the meta-analysis, 14 out of 23 studies with ≤ 100 subjects as sample size; recommending the need for other studies to confirm these findings or perhaps to significantly impact confidence in the influence evaluation.

Meta-analysis is a methodology adapted to statistically pool and study the findings from several independent randomized normal oral diet-led trials (42). Surgery is the foundation of a multimodal managing approach for a limited local region upper gastrointestinal cancer. European Society for Clinical Nutrition and Metabolism guidelines on clinical nutrition in surgery endorsed that nasojejunal feeding tube or needle catheter jejunostomy is considered for malnutrition subjects who suffered from major upper gastrointestinal surgeries (16). Furthermore, a systematic review by Yan recommended that enteral nutrition is favored in gastrointestinal cancer subjects after surgery (43). Moreover, guidelines on nutrition in cancer subjects endorsed the maintenance of nutrition treatment after hospital discharge for subjects who do not meet their needs *via* the oral method (44, 45). Lately, the European Society for Clinical Nutrition and Metabolism guideline on home enteral nutrition suggested that gastrointestinal cancer subjects at risk of malnutrition must consider oral nutritional supplements or home enteral nutrition before hospital discharge (20). In clinical practice, many subjects select to keep the nasojejunal feeding tube at hospital discharge. Choi et al. reported that 90% of their gastro-esophageal cancer subjects used a nasojejunal feeding tube after surgery, and 75% of the subjects used nasojejunal feeding tubes for home enteral nutrition after hospital discharge (46). Moreover, many hospitals follow upper gastrointestinal cancer subjects with oral nutritional supplements after hospital discharge (17, 18, 26). Malnutrition and weight loss are major problems after surgery in upper gastrointestinal subjects. Earlier randomized clinical trials and meta-analyses have reported a weight loss of up to 20% in 6 months after surgery in upper gastrointestinal subjects (6). Weight loss is the more common sign of malnutrition, and there is substantial indication that postoperative malnutrition outcomes in protein catabolism and wound healing delay, and is an independent marker of higher problems and poor prognosis between subjects who suffer upper gastrointestinal cancer surgery (47). Almost 72% of subjects can get only up to 85% of the necessary calories by oral intake at hospital discharge after upper gastrointestinal resection (5). Earlier studies reported that home enteral nutritional support after hospital discharge can supplement the everyday needs of subjects that cannot take normal oral food (26, 35). Though, the energy supplemented by home enteral nutritional support effect on weight loss in postoperative subjects with upper gastrointestinal cancers is still not consistent and needs further studies.

Subjects getting home enteral nutrition might experience nasojejunal tube-related problems, e.g., tube blocking, tube movement, and unintentional nasojejunal tube removal, which might lead to an early end of home enteral nutrition (48). Therefore, it is vital to evaluate the safety of home enteral nutritional support. This meta-analysis indicates that there was no significant difference in the complications among home enteral nutritional support and the normal oral diet, showing the safety of home enteral nutritional support. The upcoming studies should consider grouping subjects according to their nutritional status at hospital discharge. Quality of life is debatably one of the most vital criteria in assessing the success of the surgery (49). After upper gastrointestinal resection, subjects suffer from poor quality of life, which is associated with reduced physical function and symptoms, e.g., appetite loss, vomiting, fatigue, and sleep disturbance (50). Due to these problems, a number of subjects could not stand a complete treatment approach of neoadjuvant or adjuvant therapy (51), and poor quality of life has been reported to be an independent negative prognostic factor for subject death (52). Therefore, improving the postoperative physical status and decreasing symptoms is critical. Healthcare costs are also vital evidence of home enteral nutritional support. It was found that the healthcare costs of home enteral nutritional support are higher than the normal oral diet, chiefly related to the cost of enteral nutrition agents (53). However, we could not evaluate it since studies did adjust or outline this factor.

This meta-analysis showed the relationship between the influences of home enteral nutritional support compared with a normal oral diet in postoperative subjects with upper gastrointestinal cancer resection. However, further studies are needed to validate these potential associations. In addition, further studies are needed to deliver a clinically meaningful difference in the results. This was suggested in other meta-analyses which showed similar effects (53–61). This needs additional examination and clarification because no clear reasoning was found to clarify these outcomes. Well-designed clinical trials are required to evaluate these factors with the blend of diverse ages, gender, and ethnicity; as our meta-analysis study could not answer whether these factors are related to the outcomes. In summary, the home enteral nutritional support had a significantly higher quality of life, better body weight change, higher albumin, and higher pre-albumin compared to a normal oral diet in subjects with upper gastrointestinal cancer resection. However, home enteral nutritional support had no significant impact on the hemoglobin and complications compared to the normal oral diet in subjects with upper gastrointestinal cancer resection.

### Limitations

There may be a collection bias in this meta-analysis since several studies found were excluded from the meta-analysis. Though, the studies excluded did not satisfy the inclusion criteria of the meta-analysis. Furthermore, we could not decide if the results were linked to age, gender, ethnicity, overall satisfaction, the need for rehospitalization, the coverage of energy, and protein intake or not. The study designed to assess the relationship between the influence of home enteral nutritional support and normal oral diet on the outcomes of subjects with upper gastrointestinal cancer resection was depending on data from former studies, which may result in bias brought by incomplete details. The meta-analysis was depending on 23 studies; 14 studies of them were small, ≤ 100. Features comprising the age, gender, obedience, and ethnicity of subjects were also likely bias-encouraging features. Several unpublished studies and lost data may result in a pooled influence bias. Subjects were using diverse chief pharmacological medicines, treatment schedules, doses, and healthcare schemes. The length of home enteral nutritional support and normal oral diet treatment of the included studies were varying. The comprised studies did not sufficiently assess the hospital costs of the subjects studied, which is a vital result.

## Conclusions

Home enteral nutritional support had a significantly higher quality of life, better body weight change, higher albumin, and higher pre-albumin compared to the normal oral diet in subjects with upper gastrointestinal cancer resection. However, home enteral nutritional support had no significant impact on the hemoglobin and complications compared to the normal oral diet in subjects with upper gastrointestinal cancer resection. However, the analysis of outcomes should be done with consideration because of the low sample size of some of the selected studies found for the meta-analysis; recommending the need for added studies to confirm these results or perhaps to significantly influence confidence in the effect evaluation. More studies are essential to confirm these outcomes.

## Data Availability Statement

The original contributions presented in the study are included in the article/supplementary material, further inquiries can be directed to the corresponding author.

## Author Contributions

YM: conception and design. FL, XP, SZ, RR, and GC: collection and assembly of data. All authors administrative support, provision of study materials or patients, data analysis and interpretation, manuscript writing, and final approval of manuscript.

## Conflict of Interest

The authors declare that the research was conducted in the absence of any commercial or financial relationships that could be construed as a potential conflict of interest.

## Publisher's Note

All claims expressed in this article are solely those of the authors and do not necessarily represent those of their affiliated organizations, or those of the publisher, the editors and the reviewers. Any product that may be evaluated in this article, or claim that may be made by its manufacturer, is not guaranteed or endorsed by the publisher.

## References

[1] Bray F Ferlay J Soerjomataram I Siegel RL Torre LA Jemal A. Global cancer statistics 2018: GLOBOCAN estimates of incidence and mortality worldwide for 36 cancers in 185 countries. CA Cancer J Clin. (2018) 68:394–424. 10.3322/caac.21492 30207593

[2] Martin L Lagergren P. Risk factors for weight loss among patients surviving 5 years after esophageal cancer surgery. Ann Surg Oncol. (2015) 22:610–6. 10.1245/s10434-014-3973-2 25120247

[3] Attar A Malka D Sabaté J Bonnetain F Lecomte T Aparicio T . Malnutrition is high and underestimated during chemotherapy in gastrointestinal cancer: an AGEO prospective cross-sectional multicenter study. Nutr Cancer. (2012) 64:535–42. 10.1080/01635581.2012.670743 22494155

[4] Takeuchi H Miyata H Gotoh M Kitagawa Y Baba H Kimura W . A risk model for esophagectomy using data of 5354 patients included in a Japanese nationwide web-based database. Ann Surg. (2014) 260:259–66. 10.1097/SLA.0000000000000644 24743609

[5] Matsuoka M Iijima S. Consideration of nutritional support for decreased caloric intake in patients with severe weight loss after esophageal cancer surgery. Gan to Kagaku Ryoho Cancer Chemother. (2019) 46:132–4. 31189837

[6] Baker M Halliday V Williams RN Bowrey DJ. A systematic review of the nutritional consequences of esophagectomy. Clin Nutr. (2016) 35:987–94. 10.1016/j.clnu.2015.08.010 26411750 PMC5410167

[7] Greene CL DeMeester SR Worrell SG Oh DS Hagen JA DeMeester TR. Alimentary satisfaction, gastrointestinal symptoms, and quality of life 10 or more years after esophagectomy with gastric pull-up. J Thorac Cardiovasc Surg. (2014) 147:909–14. 10.1016/j.jtcvs.2013.11.004 24332098

[8] Djärv T Blazeby JM Lagergren P. Predictors of postoperative quality of life after esophagectomy for cancer. J Clin Oncol. (2009) 27:1963–8. 10.1200/JCO.2008.20.5864 19289614

[9] Mortensen K Nilsson M Slim K Schäfer M Mariette C Braga M . Consensus guidelines for enhanced recovery after gastrectomy. J Br Surg. (2014) 101:1209–29. 10.1002/bjs.9582 25047143

[10] Low DE Allum W De Manzoni G Ferri L Immanuel A Kuppusamy M . Guidelines for perioperative care in esophagectomy: enhanced recovery after surgery (ERAS®) society recommendations. World J Surg. (2019) 43:299–330. 10.1007/s00268-018-4786-4 30276441

[11] Cederholm T Barazzoni R Austin P Ballmer P Biolo G Bischoff SC . ESPEN guidelines on definitions and terminology of clinical nutrition. Clinical nutrition. (2017) 36:49–64. 10.1016/j.clnu.2016.09.004 27642056

[12] Bowrey DJ Baker M Halliday V Thomas AL Pulikottil-Jacob R Smith K . A randomised controlled trial of six weeks of home enteral nutrition versus standard care after oesophagectomy or total gastrectomy for cancer: report on a pilot and feasibility study. Trials. (2015) 16:1–12. 10.1186/s13063-015-1053-y 26590903 PMC4654827

[13] Zhou X Li B Chen A Xiang J. Delayed retention of jejunostomy tube in the recovery of gastric cancer patients. Hainan Med J. (2015) 26:576e.7.

[14] Xu X. Therapeutic evaluation of postoperative gastric cancer patients with home enteral nutrition support. Jilin: Jilin Univ. (2015) 52.

[15] Gavazzi C Colatruglio S Valoriani F Mazzaferro V Sabbatini A Biffi R . Impact of home enteral nutrition in malnourished patients with upper gastrointestinal cancer: a multicentre randomised clinical trial. Eur J Cancer. (2016) 64:107–12. 10.1016/j.ejca.2016.05.032 27391922

[16] Weimann A Braga M Carli F Higashiguchi T Hübner M Klek S . ESPEN guideline: clinical nutrition in surgery. Clin Nutr. (2017) 36:623–50. 10.1016/j.clnu.2017.02.013 28385477

[17] Ida S Hiki N Cho H Sakamaki K Ito S Fujitani K . Randomized clinical trial comparing standard diet with perioperative oral immunonutrition in total gastrectomy for gastric cancer. J Br Surg. (2017) 104:377–83. 10.1002/bjs.10417 28072447

[18] Liu X Xiao H Zhang N Chang J Yan W. Clinical application of oral nutritional supplementation (ONS) on reducing adverse reactions of chemotherapy in patients with gastric cancer after radical gastrectomy. J Dig Oncol. (2018) 10:238e.41.

[19] Jordan S Philpin S Warring J Cheung WY Williams J. Percutaneous endoscopic gastrostomies: the burden of treatment from a patient perspective. J Adv Nurs. (2006) 56:270–81. 10.1111/j.1365-2648.2006.04006.x 17042806

[20] Bischoff SC Austin P Boeykens K Chourdakis M Cuerda C Jonkers-Schuitema C . ESPEN guideline on home enteral nutrition. Clin Nutr. (2020) 39:5–22. 10.1016/j.clnu.2019.04.022 31255350

[21] Stroup DF Berlin JA Morton SC Olkin I Williamson GD Rennie D . Meta-analysis of observational studies in epidemiology: a proposal for reporting. Jama. (2000) 283:2008–12. 10.1001/jama.283.15.2008 10789670

[22] Gupta A Das A Majumder K Arora N Mayo HG Singh PP . Obesity is independently associated with increased risk of hepatocellular cancer–related mortality. Am J Clin Oncol. (2018) 41:874–81. 10.1097/COC.0000000000000388 28537989 PMC5700876

[23] Liberati A Altman DG Tetzlaff J Mulrow C Gøtzsche PC Ioannidis JP . The PRISMA statement for reporting systematic reviews and meta-analyses of studies that evaluate health care interventions: explanation and elaboration. J Clin Epidemiol. (2009) 62:e1–e34. 10.1016/j.jclinepi.2009.06.006 19631507

[24] Collaboration C. RoB 2: A revised Cochrane risk-of-bias tool for randomized trials. Available online at: bias/resources/rob-2-revised-cochrane-risk-bias-tool-randomized-trials. (Accessed December 6, 2019).

[25] Higgins JP Thompson SG Deeks JJ Altman DG. Measuring inconsistency in meta-analyses. Bmj. (2003) 327:557–60. 10.1136/bmj.327.7414.557 12958120 PMC192859

[26] Imamura H Nishikawa K Kishi K Inoue K Matsuyama J Akamaru Y . Effects of an oral elemental nutritional supplement on post-gastrectomy body weight loss in gastric cancer patients: a randomized controlled clinical trial. Ann Surg Oncol. (2016) 23:2928–35. 10.1245/s10434-016-5221-4 27084538

[27] Hatao F Chen KY Wu JM Wang MY Aikou S Onoyama H . Randomized controlled clinical trial assessing the effects of oral nutritional supplements in postoperative gastric cancer patients. Langenbeck's Arch Surg. (2017) 402:203–11. 10.1007/s00423-016-1527-8 27807617

[28] Zeng J Hu J Chen Q Feng J. Home enteral nutrition's effects on nutritional status and quality of life after esophagectomy. Asia Pac J Clin Nutr. (2017) 26:804–10. 28802289 10.6133/apjcn.112016.07

[29] Froghi F Sanders G Berrisford R Wheatley T Peyser P Rahamim J . A randomised trial of post-discharge enteral feeding following surgical resection of an upper gastrointestinal malignancy. Clinical Nutrition. (2017) 36:1516–9. 10.1016/j.clnu.2016.10.022 27842926

[30] Ren H Su Z Yang Y Zhao Y Wang X Di H. The feasibility of family enteral nutrition after total gastrectomy for gastric cancer. Food Nutr China. (2017) 24:61e.3.

[31] Cui H Yang X Tang D Zhou X Ding R Zhu M . Effect of oral nutritional supplementation on nutritional status and quality of life in patients with gastric cancer after operation (23 cases RCT observations). Chin J Clin Nutr. (2017) 25:183–8.

[32] Cui H Yang X Tang D Zhou X Ding R Zhu M . Effect of oral nutritional supplementation on nutritional status and quality of life in patients with gastric cancer after operation (23 cases RCT observations). Chin J Clin Nutr. (2017) 25:183–188.

[33] Zhang M Zhu X Ding C Kong L Chen Y. Study on the feasibility of enteral nutrition after total gastrectomy for gastric cancer 4 weeks hom. Chin J Surg Oncol. (2017) 9:113e.6.

[34] Kong SH Lee HJ Na JR Kim WG Han DS Park SH . Effect of perioperative oral nutritional supplementation in malnourished patients who undergo gastrectomy: a prospective randomized trial. Surgery. (2018) 164:1263–70. 10.1016/j.surg.2018.05.017 30055788

[35] Liu K Ji S Xu Y Diao Q Shao C Luo J . Safety, feasibility, and effect of an enhanced nutritional support pathway including extended preoperative and home enteral nutrition in patients undergoing enhanced recovery after esophagectomy: a pilot randomized clinical trial. Dis Esophagus. (2020) 33:doz030. 10.1093/dote/doz030 31329828

[36] Li XK Cong ZZ Wu WJ Ji SG Zhou H Liu KC . Efficacy of 4 wk of home enteral feeding supplementation after esophagectomy on immune function: a randomized controlled trial. Nutrition. (2020) 77:110787. 10.1016/j.nut.2020.110787 32438300

[37] Yang X Zhu M Xiu D Yang Y Yang G Hu W . Effect of an oral nutritional supplementation on nutritional status and quality of life in patients with colorectal cancer and postoperative adjuvant chemotherapy: A multi-center prospective randomized control trial. Chin J Gastrointest Surg. (2020) 23:566–71. 32521976 10.3760/cma.j.cn.441530-20190724-00287

[38] Meng Q Tan S Jiang Y Han J Xi Q Zhuang Q . Post-discharge oral nutritional supplements with dietary advice in patients at nutritional risk after surgery for gastric cancer: a randomized clinical trial. Clin Nutr. (2021) 40:40–6. 10.1016/j.clnu.2020.04.043 32563598

[39] Tan S Meng Q Jiang Y Zhuang Q Xi Q Xu J . Impact of oral nutritional supplements in post-discharge patients at nutritional risk following colorectal cancer surgery: a randomised clinical trial. Clin Nutr. (2021) 40:47–53. 10.1016/j.clnu.2020.05.038 32563599

[40] Miyazaki Y Omori T Fujitani K Fujita J Kawabata R Imamura H . Oral nutritional supplements versus a regular diet alone for body weight loss after gastrectomy: a phase 3, multicenter, open-label randomized controlled trial. Gastric Cancer. (2021) 24:1150–9. 10.1007/s10120-021-01188-3 33835329

[41] Yang F Li L Mi Y Zou L Chu X Sun A . Effectiveness of an early, quantified, modified oral feeding protocol on nutritional status and quality of life of patients after minimally invasive esophagectomy: a retrospective controlled study. Nutrition. (2021) 94:111540. 10.1016/j.nut.2021.111540 34965500

[42] Lau J Ioannidis JP Schmid CH. Summing up evidence: one answer is not always enough. Lancet. (1998) 351:123–7. 10.1016/S0140-6736(97)08468-7 9439507

[43] Yan X Zhou FX Lan T Xie CH Dai J Fu ZM . Optimal postoperative nutrition support for patients with gastrointestinal malignancy: a systematic review and meta-analysis. Clin Nutr. (2017) 36:710–21. 10.1016/j.clnu.2016.06.011 27452745

[44] Wu G Tan S. Guidelines on nutritional support in patients with tumor. Chin J Surg. (2017) 55:801–29. 29136728 10.3760/cma.j.issn.0529-5815.2017.11.001

[45] Arends J Bachmann P Baracos V Barthelemy N Bertz H Bozzetti F . ESPEN guidelines on nutrition in cancer patients. Clin Nutr. (2017) 36:11–48. 10.1016/j.clnu.2016.07.015 27637832

[46] Choi AH O'Leary MP Merchant SJ Sun V Chao J Raz DJ . Complications of feeding jejunostomy tubes in patients with gastroesophageal cancer. J Gastrointest Surg. (2017) 21:259–65. 10.1007/s11605-016-3297-6 27785689 PMC5568416

[47] Heneghan HM Zaborowski A Fanning M McHugh A Doyle S Moore J . Prospective study of malabsorption and malnutrition after esophageal and gastric cancer surgery. Ann Surg. (2015) 262:803–8. 10.1097/SLA.0000000000001445 26583669

[48] Kingma BF Steenhagen E Ruurda JP van Hillegersberg R. Nutritional aspects of enhanced recovery after esophagectomy with gastric conduit reconstruction. J Surg Oncol. (2017) 116:623–9. 10.1002/jso.24827 28968919

[49] Syn NL Wee I Shabbir A Kim G So JB. Pouch versus no pouch following total gastrectomy: meta-analysis of randomized and non-randomized studies. Ann Surg. (2019) 269:1041–53. 10.1097/SLA.0000000000003082 31082900

[50] Gannon J Guinan E Doyle S Beddy P Reynolds J Hussey J. Reduced fitness and physical functioning are long-term sequelae after curative treatment for esophageal cancer: a matched control study. Dis Esophagus. (2017) 30:1–7. 10.1093/dote/dox018 28575241

[51] Smalley SR Benedetti JK Haller DG Hundahl SA Estes NC Ajani JA . Updated analysis of SWOG-directed intergroup study 0116: a phase III trial of adjuvant radiochemotherapy versus observation after curative gastric cancer resection. J Clin Oncol. (2012) 30:2327. 10.1200/JCO.2011.36.7136 22585691 PMC4517071

[52] McKernan M McMillan DC Anderson JR Angerson WJ Stuart RC. The relationship between quality of life (EORTC QLQ-C30) and survival in patients with gastro-oesophageal cancer. Br J Cancer. (2008) 98:888–93. 10.1038/sj.bjc.6604248 18268490 PMC2266859

[53] Xueting H Meng Y Yuqing C Yutong H Lihong Q June Z. Home enteral nutrition and oral nutritional supplements in postoperative patients with upper gastrointestinal malignancy: a systematic review and meta-analysis. Clin Nutr. (2021) 40:3082–93. 10.1016/j.clnu.2020.11.023 33279310

[54] Rinninella E Cintoni M Raoul P Pozzo C Strippoli A Bria E . Effects of nutritional interventions on nutritional status in patients with gastric cancer: A systematic review and meta-analysis of randomized controlled trials. Clin Nutr. ESPEN. (2020) 38:28–42. 10.1016/j.clnesp.2020.05.007 32690170

[55] Ding J Sun B Song P Liu S Chen H Feng M . The application of enhanced recovery after surgery (ERAS)/fast-track surgery in gastrectomy for gastric cancer: a systematic review and meta-analysis. Oncotarget. (2017) 8:75699. 10.18632/oncotarget.18581 29088903 PMC5650458

[56] Reece L Hogan S Allman-Farinelli M Carey S. Oral nutrition interventions in patients undergoing gastrointestinal surgery for cancer: a systematic literature review. Support Care Cancer. (2020) 28:5673–91. 10.1007/s00520-020-05673-w 32815021

[57] Zhao B Luo Y Lu R. Evaluate the effect of home enteral nutrition compared with oral diet after the hospital discharge of subjects with esophagectomy of esophageal cancer: a systemic review and meta-analysis. J Food Nutr Res. (2021) 9:571–8. 10.12691/jfnr-9-11-3

[58] Liu L Wang YC Liu QW Zhong JD Li JB Wu XD . Home enteral nutrition after esophagectomy for esophageal cancer: a systematic review and meta-analysis. Medicine. (2020) 99. 10.1097/MD.0000000000021988 PMC7478745 32899043

[59] Chen X Yang K Zhang X Li K. Meta-analysis of preoperative oral nutritional supplements for patients with gastric cancer: east Asian experience. Eur J Clin Nutr. (2020) 74:991–1000. 10.1038/s41430-019-0483-0 31371794

[60] Mulazzani GE Corti F Della Valle S Di Bartolomeo M. Nutritional support indications in gastroesophageal cancer patients: from perioperative to palliative systemic therapy. A comprehensive review of the last decade. Nutrients. (2021) 13:2766. 10.3390/nu13082766 34444926 PMC8400027

[61] Mei LX Wang YY Tan X Chen Y Dai L Chen MW. Is it necessary to routinely perform feeding jejunostomy at the time of esophagectomy? A systematic review and meta-analysis. Dis Esophagus. (2021) 34. 10.1093/dote/doab017 33884417

